# Supplementary material for: Single-cell transcriptome profiling of the vaginal wall in women with severe anterior vaginal prolapse
Source: Nat Commun. 2021 Jan 4;12:87. doi: 10.1038/s41467-020-20358-y (PMC7782707; doi:10.1038/s41467-020-20358-y)
Supplement: Supplementary file 1 — Supplementary Information [file 41467_2020_20358_MOESM1_ESM.pdf]

Supplementary Information

Single-cell transcriptome profiling of the vaginal wall in women with severe anterior vaginal prolapse

Li et al.

Supplementary Figure 1

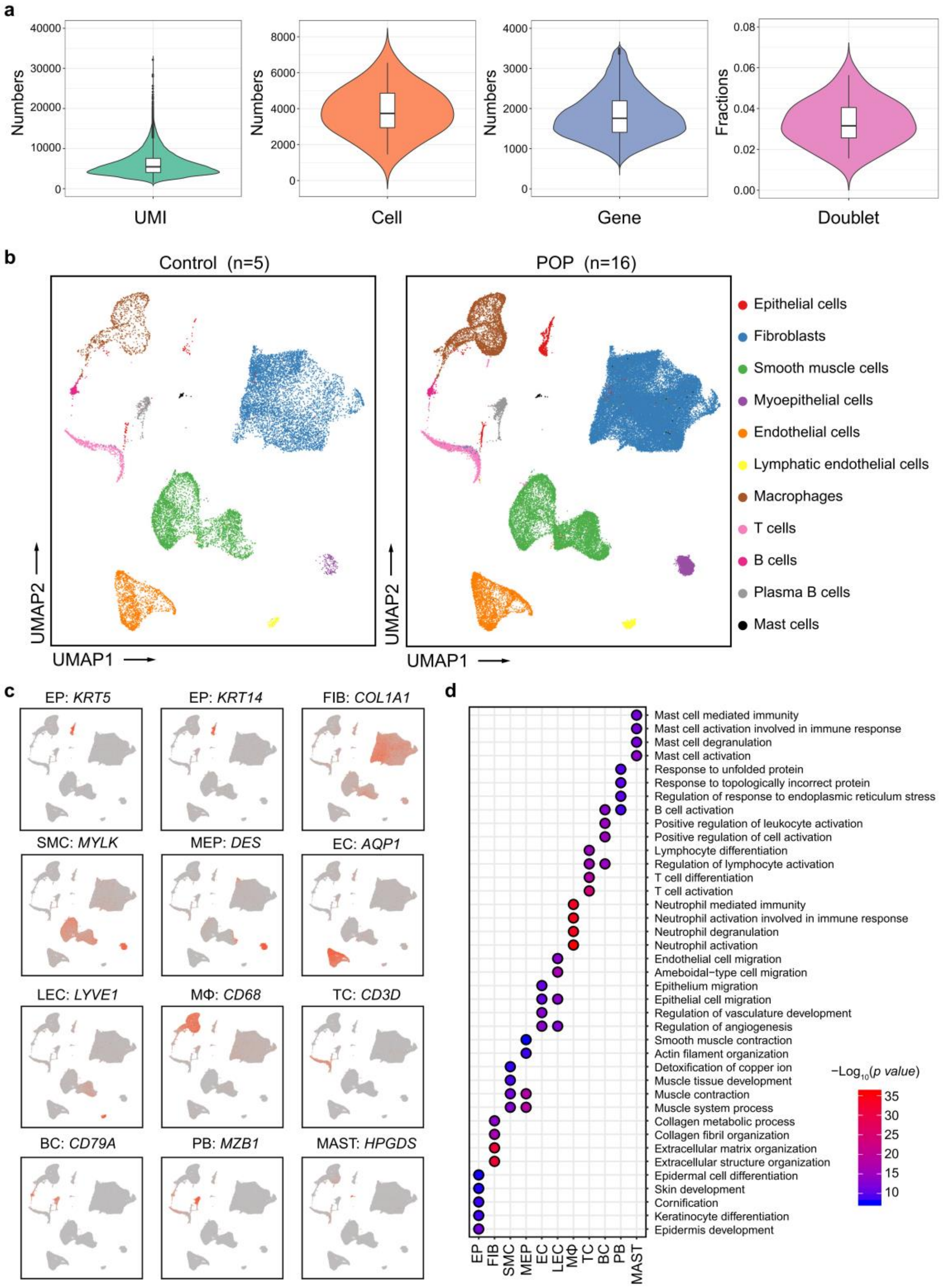

Supplementary Fig. 1 Primary data analysis and distribution of clusters across different samples. a Violin plot showing the number of UMI, cell, genes and ratio of doublet across different samples (Control, n=5 patients; POP, n=16 patients). The horizontal line within each box represents the median, and the top and bottom of each

box indicate the 75th and 25th percentile. Two-sided Wilcoxon rank-sum test was applied to test the significance of the gene expression with p-value < 0.05. b UMAP plot showing the different cell types in control (n=5) and POP (n=16) samples. c UMAP plot showing expression of canonic markers for each cell type. d Dot plot showing the GO enrichment results of differentially expression genes in each cell type. EP, epithelial cell; FIB, fibroblasts; SMC, smooth muscle cells; MEP, myoepithelial cells; EC, endothelial cells; LEC, lymphatic endothelial cells; MΦ, macrophages; TC, T cells; BC, B cells; PB, plasma B cells; MAST, mast cells.

Supplementary Figure 2

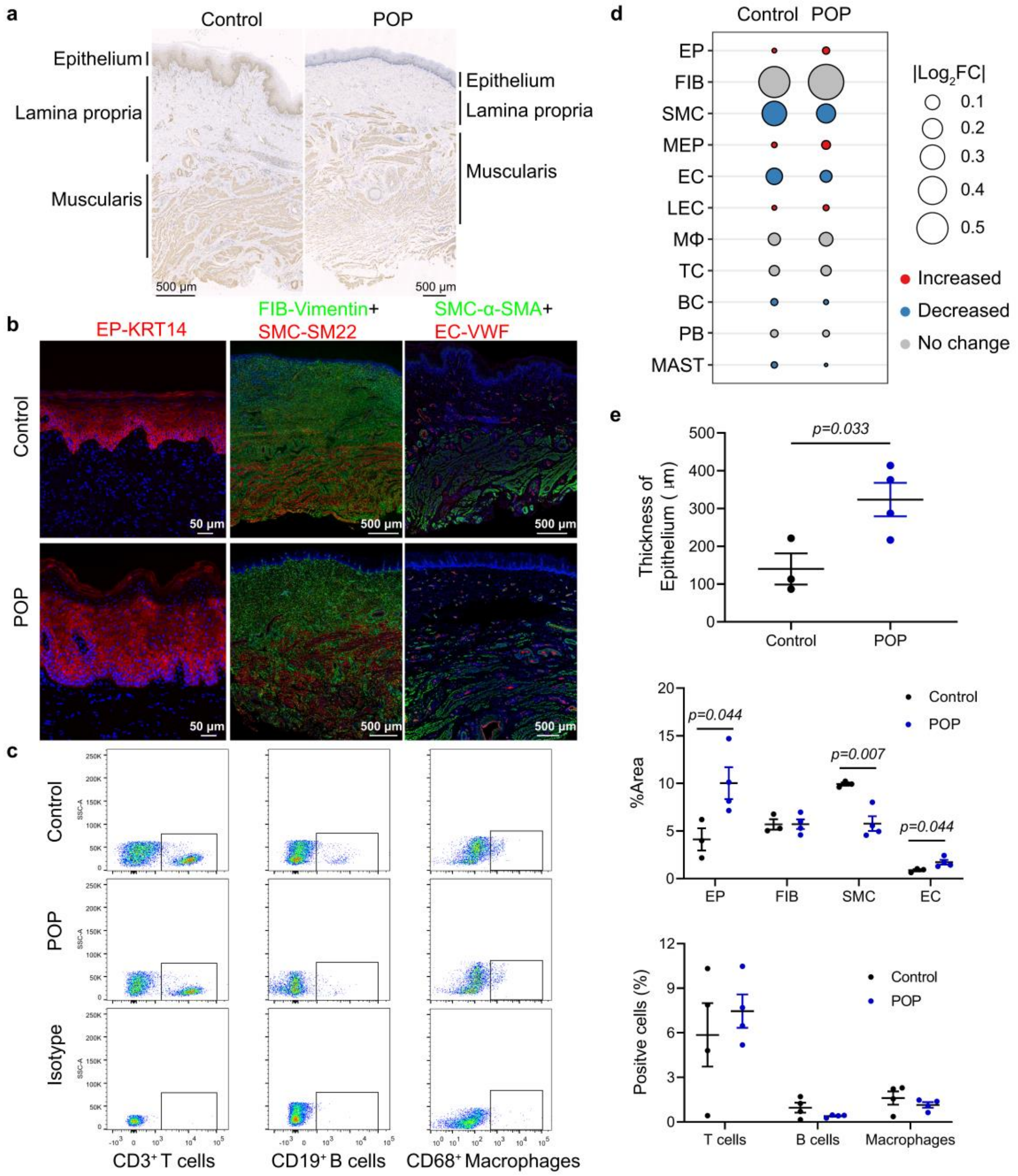

Supplementary Fig. 2 Histological Characterizations of the main cell types and changes in the composition of the main cell types. a IHC staining for α-SMA in control and POP samples. Representative images were shown. b Immunofluorescence staining for epithelial cells, fibroblasts, smooth muscle cells and endothelial cells. Control, n=3 patients; POP, n=4 patients. c Flow cytometric analysis of T cells, B cells and macrophages. Antibodies of CD3, CD19, and CD68 were detected in each sample, respectively. The corresponding isotypes were used for gating as negative population.

d Relative changes in cell ratios in control and POP samples according to computational analysis. e Statistical analysis of the positive staining cells. The epithelium thickness was measured from H&E staining in control and POP samples. Areas of positive regions were measured from immunostaining images of epithelial cells (KRT14), fibroblasts (Vimentin), smooth muscle cells (α-SMA) and endothelial cells (VWF). For histological analysis, control, n=3 patients; POP, n=4 patients. For flow cytometric analysis, control, n=4 patients; POP, n=4 patients. Data are presented as the means ± SEM; *P* values were determined by two-tailed unpaired Student's t test. EP, epithelial cell; FIB, fibroblasts; SMC, smooth muscle cells; MEP, myoepithelial cells; EC, endothelial cells; LEC, lymphatic endothelial cells; MΦ, macrophages; TC, T cells; BC, B cells; PB, plasma B cells; MAST, mast cells.

Supplementary Figure 3

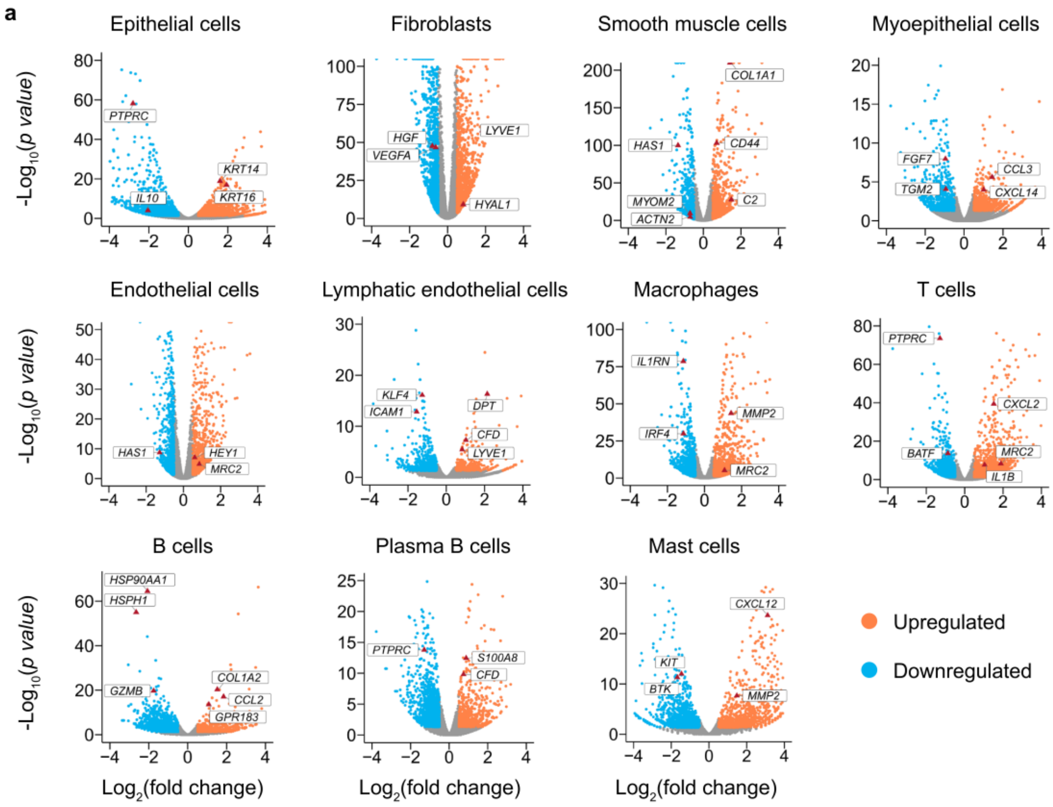

b

Gene number	EP	FIB	SMC	MEP	EC	LEC	MΦ	TC	BC	PB	MAST
Upregulated	1331	1389	1177	801	1467	332	1634	1761	561	698	1144
Downregulated	1937	1349	613	747	913	652	1291	1421	1631	1519	1329

Supplementary Fig. 3 Differential gene expression in different cell types between control and POP samples. a Volcano plot showing the differential expressed genes (DEGs) in each cell type, in which some representative genes were marked with red triangle. b The number of DEGs in each cell type was shown. The asterisk represents the cell types in which the upregulated genes was lower than the number of downregulated genes. EP, epithelial cell; FIB, fibroblasts; SMC, smooth muscle cells; MEP, myoepithelial cells; EC, endothelial cells; LEC, lymphatic endothelial cells; MΦ, macrophages; TC, T cells; BC, B cells; PB, plasma B cells; MAST, mast cells.

Supplementary Figure 4

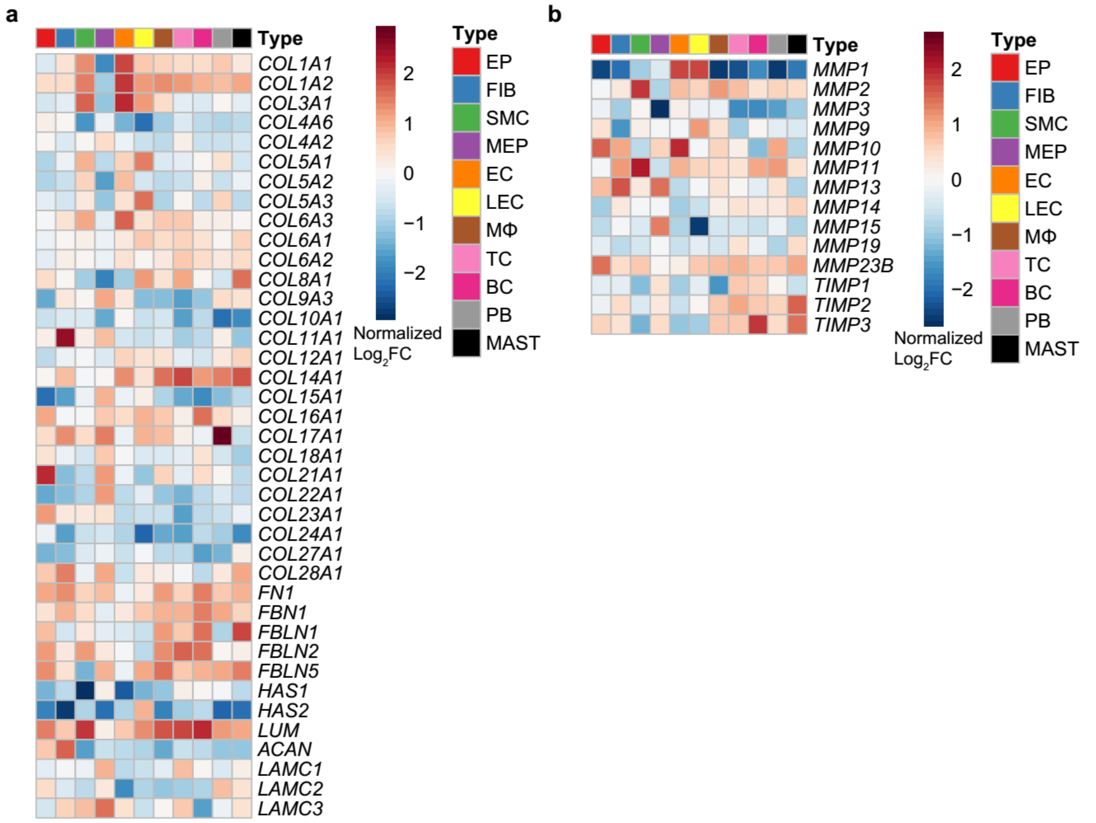

Supplementary Fig. 4 The expression changes of representative extracellular matrix related genes in the cell type level. a The expression changes of representative collagen family members and other extracellular matrix related genes in different cell types in POP samples than that in control samples. b The expression changes of representative matrix metalloproteinase family members and its inhibitor in different cell types in POP samples than that in control samples. EP, epithelial cell; FIB, fibroblasts; SMC, smooth muscle cells; MEP, myoepithelial cells; EC, endothelial cells; LEC, lymphatic endothelial cells; MΦ, macrophages; TC, T cells; BC, B cells; PB, plasma B cells; MAST, mast cells.

Supplementary Figure 5

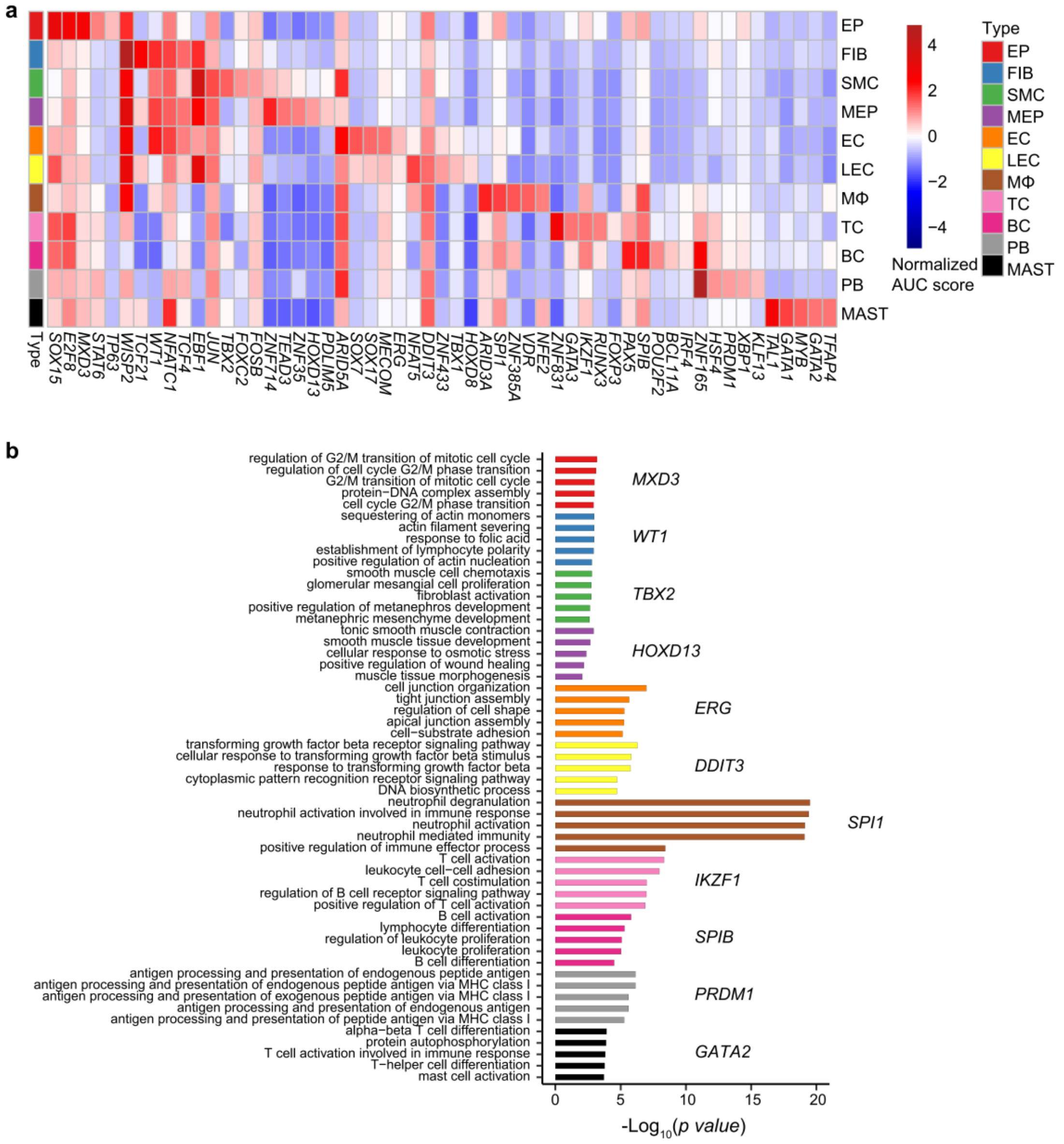

Supplementary Fig. 5 Cell type-specific TFs and target genes on control samples. a Heatmap showing the activity of top 5 cell type-specific TFs (columns) in each cell type (rows) as identified by SCENIC. b Bar plots indicating the GO enrichment results of representative TFs and corresponding target genes. EP, epithelial cell; FIB, fibroblasts; SMC, smooth muscle cells; MEP, myoepithelial cells; EC, endothelial cells; LEC, lymphatic endothelial cells; MΦ, macrophages; TC, T cells; BC, B cells; PB, plasma B cells; MAST, mast cells.

Supplementary Figure 6

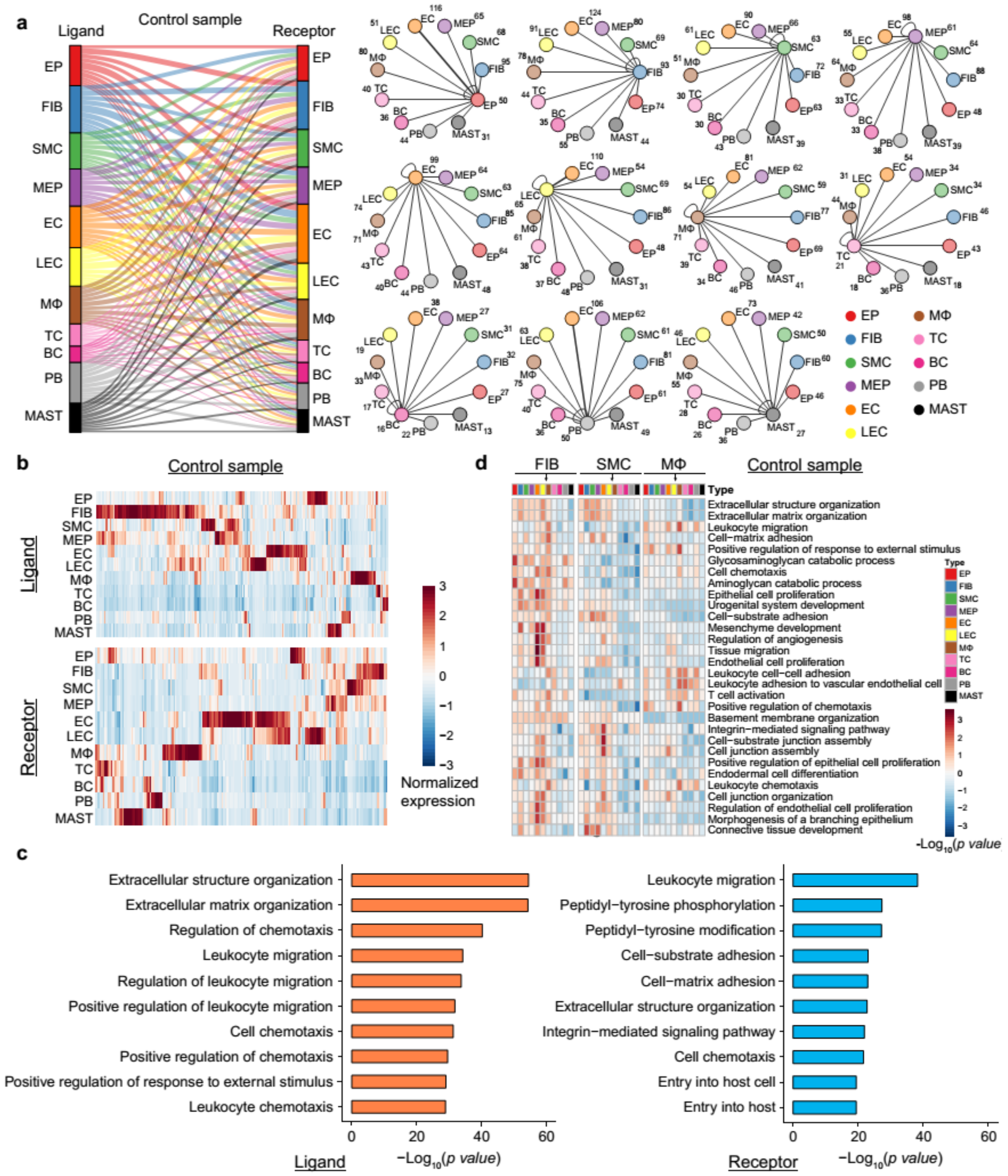

Supplementary Fig. 6 The feature of ligand-receptor interaction pairs in control samples. a Sankey plot showing the cell-cell interaction strength across different cell types (left panel). Detailed view of the number of pairs from one cell type to another (right panel). The line thickness indicates the number of ligand-receptor pairs. b The relative expression level of representative ligands and receptors in each cell type. c Bar plots displaying the GO enrichment results for expressed ligands and receptors respectively. Fisher's exact tests (two-side) were performed with *p*-value <0.05 was

defined as statistically significant. d Heatmap showing the GO enrichment results for ligand-receptor pairs in three major cell types (fibroblasts, smooth muscle cells and macrophages). EP, epithelial cell; FIB, fibroblasts; SMC, smooth muscle cells; MEP, myoepithelial cells; EC, endothelial cells; LEC, lymphatic endothelial cells; MΦ, macrophages; TC, T cells; BC, B cells; PB, plasma B cells; MAST, mast cells.

Supplementary Figure 7

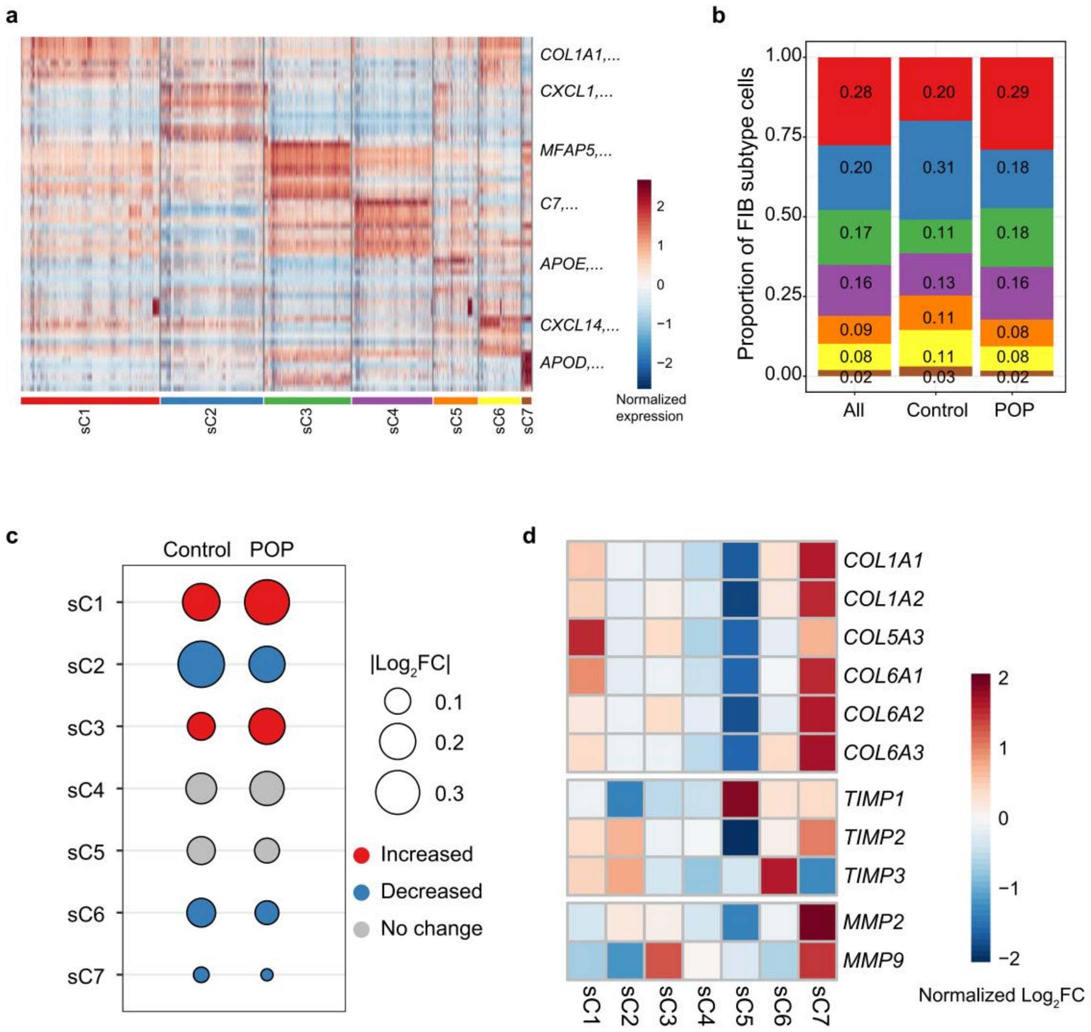

Supplementary Fig. 7 Subclustering of fibroblasts. a Heatmap showing expression signatures of top 10 DEGs in each subtype. B Bar plots showing the percentage of 7 fibroblast subtypes in control and POP samples. c Dot plot displaying relative changes in cell ratios in each subtype across control and POP samples (red, increased; blue, decreased; grey, no significant change). d The expression changes of representative extracellular matrix related genes in each subtype in POP samples than that in control samples.

Supplementary Figure 8

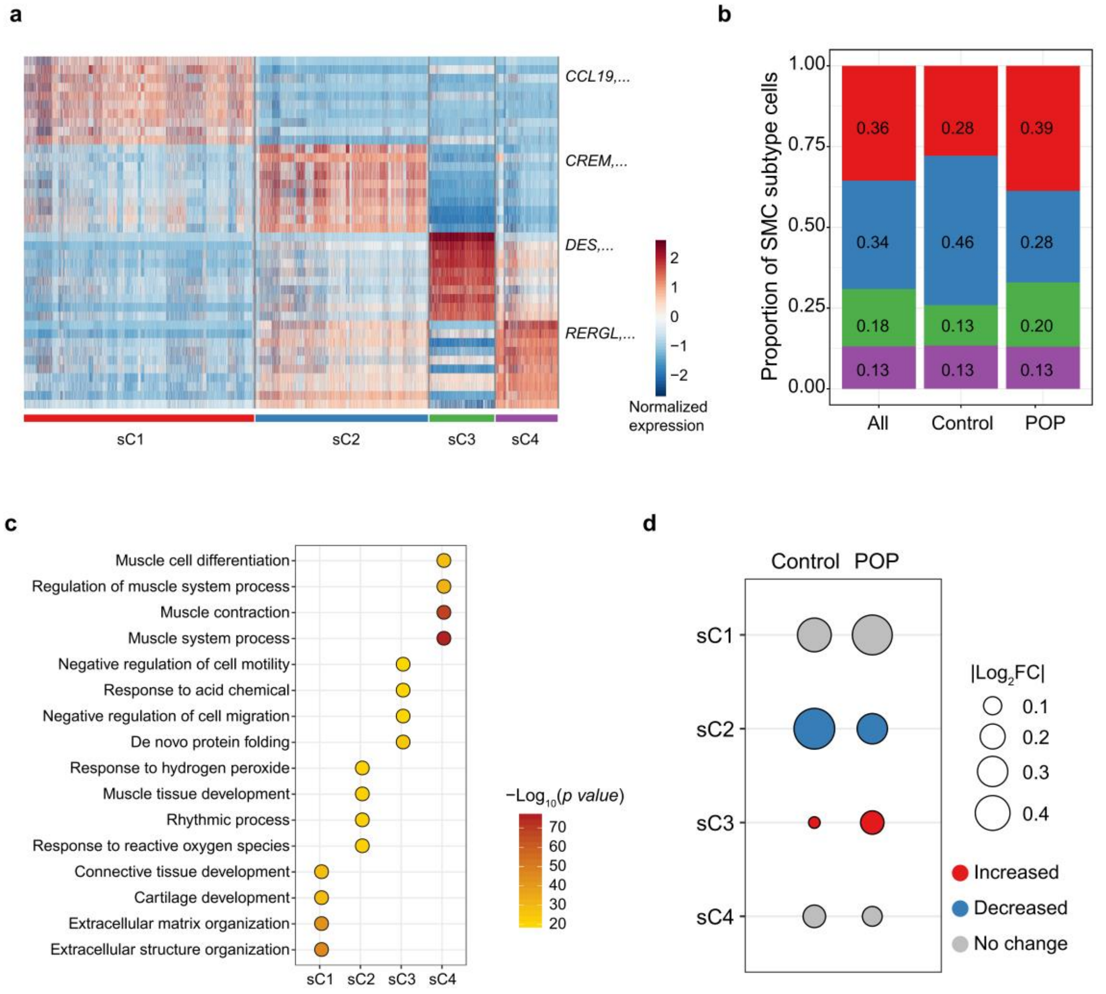

Supplementary Fig. 8 Subclustering of smooth muscle cells. a Heatmap showing expression signatures of top 10 DEGs in each subtype. b Bar plots showing the percentage of 4 smooth muscle cells subtypes in control and POP samples. c GO enrichment for DEGs in each subtype. d Dot plot displaying relative changes in cell ratios in each subtype across control and POP samples (red, increased; blue, decreased; grey, no significant change).

Supplementary Figure 9

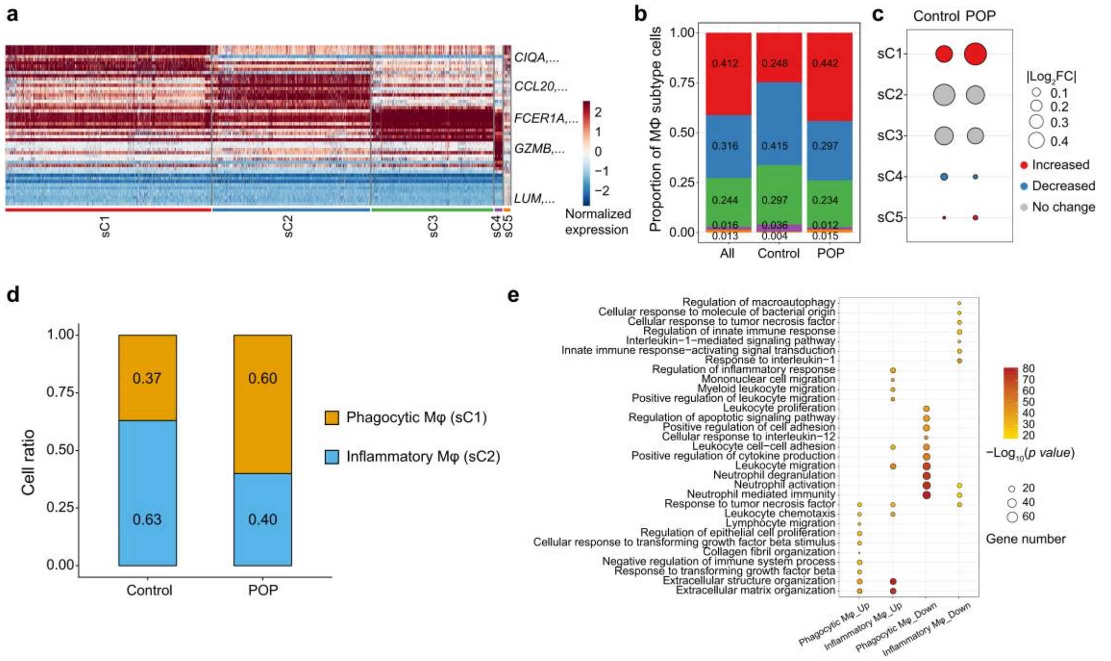

Supplementary Fig. 9 Subclustering of macrophages. a Heatmap showing expression signatures of top 10 DEGs in each subtype. b Bar plots showing the percentage of 5 macrophages subtypes in control and POP samples. c Dot plot displaying relative changes in cell ratios in each subtype across control and POP samples (red, increased; blue, decreased; grey, no significant change). d Bar plot showing the percentage of phagocytic MΦ and inflammatory MΦ cells in control and POP samples. e GO enrichment of upregulated or downregulated genes in two subtypes in POP samples. Control, n=5 patients; POP, n=16 patients.

Supplementary Figure 10

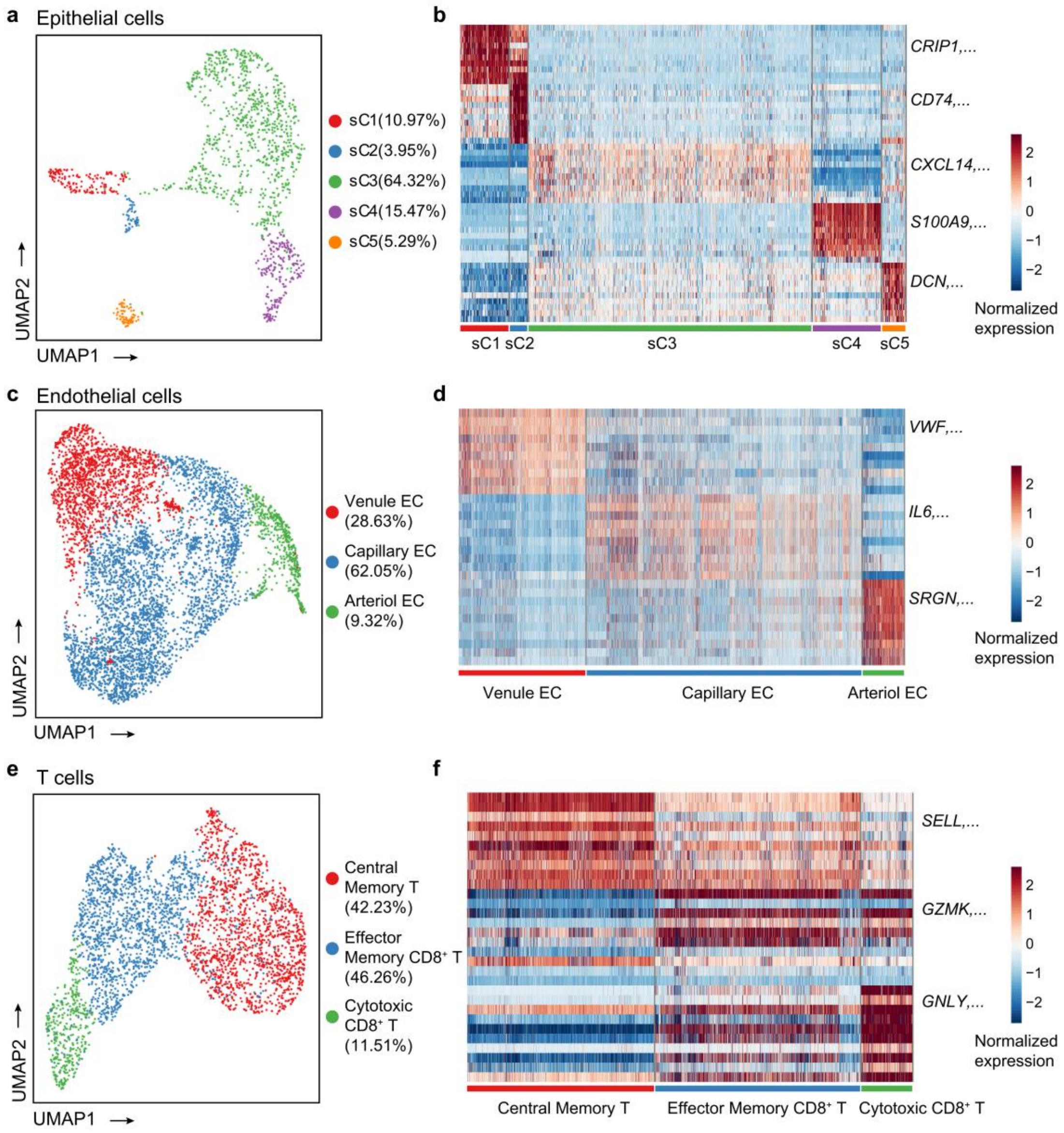

Supplementary Fig. 10 Subclustering of epithelial cells, endothelial cells and T cells. a UMAP plot showing 5 subtypes in epithelial cells. b Heatmap showing expression signatures of top 10 DEGs in each subtype of epithelial cells. c UMAP plot showing 3 subtypes in endothelial cells. d Heatmap showing expression signatures of top 10 DEGs in each subtype of endothelial cells. e UMAP plot showing 3 subtypes of T cells. f Heatmap showing expression signatures of top 10 DEGs in each subtype of T cells.
