# Supplementary material for: Single-cell transcriptome profiling of the vaginal wall in women with severe anterior vaginal prolapse
Source: Nat Commun. 2021 Jan 4;12:87. doi: 10.1038/s41467-020-20358-y (PMC7782707; doi:10.1038/s41467-020-20358-y)
Supplement: Supplementary file 3 — Description of Additional Supplementary Files [file 41467_2020_20358_MOESM3_ESM.pdf]

Description of Additional Supplementary Files

Supplementary data 1. Clinical characteristics of POP patients and control samples profiled by sc-RNA seq and validated by staining in this study.

Supplementary data 2. Characterization of cell type in each sample.

Supplementary data 3. Cluster-specific marker genes via differential gene expression analysis.

Supplementary data 4. Differentially expressed genes (DEGs) in POP samples via bulk RNA-seq analysis.

Supplementary data 5. The gene ontology (GO) terms of upregulated and downregulated genes in bulk RNA-seq analysis.

Supplementary data 6. Differentially expressed genes (DEGs) of 11 clusters via scRNA-seq analysis.

Supplementary data 7. Subtype proportion in control and POP samples.

Supplementary data 8. Subtype proportion in each sample.
