# Supplementary material for: Single-cell transcriptome profiling of the vaginal wall in women with severe anterior vaginal prolapse
Source: Nat Commun. 2021 Jan 4;12:87. doi: 10.1038/s41467-020-20358-y (PMC7782707; doi:10.1038/s41467-020-20358-y)
Supplement: Supplementary file 12 — Reporting Summary [file 41467_2020_20358_MOESM12_ESM.pdf]

# nature research

Corresponding author(s): Yun-Gui Yang, Lan Zhu

Last updated by author(s): Nov 13, 2020

# Reporting Summary

Nature Research wishes to improve the reproducibility of the work that we publish. This form provides structure for consistency and transparency in reporting. For further information on Nature Research policies, see our Editorial Policies and the Editorial Policy Checklist.

## Statistics

For all statistical analyses, confirm that the following items are present in the figure legend, table legend, main text, or Methods section.

| n/a | Confirmed | |
|---|---|---|
| [ ] | [x] | The exact sample size ($n$) for each experimental group/condition, given as a discrete number and unit of measurement |
| [ ] | [x] | A statement on whether measurements were taken from distinct samples or whether the same sample was measured repeatedly |
| [ ] | [x] | The statistical test(s) used AND whether they are one- or two-sided<br>*Only common tests should be described solely by name; describe more complex techniques in the Methods section.* |
| [x] | [ ] | A description of all covariates tested |
| [ ] | [x] | A description of any assumptions or corrections, such as tests of normality and adjustment for multiple comparisons |
| [ ] | [x] | A full description of the statistical parameters including central tendency (e.g. means) or other basic estimates (e.g. regression coefficient) AND variation (e.g. standard deviation) or associated estimates of uncertainty (e.g. confidence intervals) |
| [ ] | [x] | For null hypothesis testing, the test statistic (e.g. $F$, $t$, $r$) with confidence intervals, effect sizes, degrees of freedom and $P$ value noted<br>*Give P values as exact values whenever suitable.* |
| [x] | [ ] | For Bayesian analysis, information on the choice of priors and Markov chain Monte Carlo settings |
| [x] | [ ] | For hierarchical and complex designs, identification of the appropriate level for tests and full reporting of outcomes |
| [x] | [ ] | Estimates of effect sizes (e.g. Cohen's $d$, Pearson's $r$), indicating how they were calculated |

*Our web collection on statistics for biologists contains articles on many of the points above.*

## Software and code

Policy information about availability of computer code

**Data collection**

No software was used for data collection.

**Data analysis**

1) Cell Ranger count (v 2.1.0) for scRNA-seq data alignment.
2) Seurat (v 3.1.2.9010) for data quality control and cell clustering.
3) DoubletFinder (v 2.0.2) to remove doublets.
4) edgeR (v3.18.1) for DEGs analysis.
5) clusterprofiler (v3.13.0) for GO enrichment.
6) fgsea (v1.8.0) for gene set enrichment with biological precession gene sets (c5.bp.v6.2.symbols.gmt).
7) pySCENIC (v0.9.15) for TFs analysis.
8) GRNboost2 (arboreto 0.1.5) for gene regulatory networks generation.
9) FastQC (version 0.11.5) for the quality check of bulk-seq sequencing.
10) HISAT2 (v2.0.5) for bulk RNA-seq data alignment.
11) featureCounts (v1.6.0) for reads count calculating.
12) FlowJo software (v.10.0.7) for flow cytometry data analysis.

Code is available on Github (https://github.com/zqyhyunbin/POP).

For manuscripts utilizing custom algorithms or software that are central to the research but not yet described in published literature, software must be made available to editors and reviewers. We strongly encourage code deposition in a community repository (e.g. GitHub). See the Nature Research guidelines for submitting code & software for further information.

## Data

Policy information about availability of data

All manuscripts must include a data availability statement. This statement should provide the following information, where applicable:

- Accession codes, unique identifiers, or web links for publicly available datasets
- A list of figures that have associated raw data
- A description of any restrictions on data availability

Raw sequencing data and processed data are available through the NCBI Gene Expression Omnibus (GEO) under accession number GSE151202 [https://www.ncbi.nlm.nih.gov/geo/query/acc.cgi?acc=GSE151202] and at the Genome Sequence Archive (GSA) with accession number HRA000136 [https://bigd.big.ac.cn/gsa-human/s/j4HFr7FQ]. These data have been deposited in the Genome Sequence Archive under project PRJCA002344. Supplementary Figure 2e contains related raw data.

# Field-specific reporting

Please select the one below that is the best fit for your research. If you are not sure, read the appropriate sections before making your selection.

- [x] Life sciences
- [ ] Behavioural & social sciences
- [ ] Ecological, evolutionary & environmental sciences

For a reference copy of the document with all sections, see nature.com/documents/nr-reporting-summary-flat.pdf

# Life sciences study design

All studies must disclose on these points even when the disclosure is negative.

| | |
|---|---|
| Sample size | Sample size for 10X Genomics scRNA-seq and bulk RNA-seq was determined by the availability of patient samples.<br>No statistical method was used for sample size calculation but it was sufficient for this proof-of-concept study.<br>In this study, we used 5 control individuals undergoing hysterectomy and 16 POP patients for single-cell sequencing, which is statistically sufficient to analyze gene expression changes between control and POP samples. |
| Data exclusions | For the scRNA-seq analysis, low quality cells from all samples were excluded by retaining cells expressed 500 to 3500 genes inclusive with mitochondrial content less than 10 percent, Doublets was excluded with default parameters by DoubletFinder. The exclusion criteria were pre-established. |
| Replication | All the experimental findings were reliably reproduced. We used 21 patient samples for single-cell sequencing, which supported the reliability of the dataset. At least two biological replicates were used for each bulk-seq analysis. For biological experiments, results were confirmed in at least three individuals. We also stated the number of repeats/replications for each experiment in the methods and indicated the detailed information of samples in supplementary data 1. |
| Randomization | The patients with POP and control patients were consecutively recruited between May 2018 and December 2019 at Peking Union Medical College Hospital. |
| Blinding | Data acquisition and analysis were performed by investigators blinded to experimental groups. |

# Reporting for specific materials, systems and methods

We require information from authors about some types of materials, experimental systems and methods used in many studies. Here, indicate whether each material, system or method listed is relevant to your study. If you are not sure if a list item applies to your research, read the appropriate section before selecting a response.

### Materials & experimental systems

| n/a | Involved in the study |
|---|---|
| [ ] | [x] Antibodies |
| [x] | [ ] Eukaryotic cell lines |
| [x] | [ ] Palaeontology and archaeology |
| [x] | [ ] Animals and other organisms |
| [ ] | [x] Human research participants |
| [x] | [ ] Clinical data |
| [x] | [ ] Dual use research of concern |

### Methods

| n/a | Involved in the study |
|---|---|
| [x] | [ ] ChIP-seq |
| [ ] | [x] Flow cytometry |
| [x] | [ ] MRI-based neuroimaging |

## Antibodies

| | |
|---|---|
| Antibodies used | Application, Name, Supplier, Catalog number, Clone name, Dilution<br>IHC/IF, Anti-alpha smooth muscle Actin, Abcam, ab32575, E184, 1:300(IHC); 1:200(IF) |

IF, Anti-TAGLN/Transgelin/SM22, Abcam, ab10135, Polyclonal, 1:500
IF,Anti-Cytokeratin 14, Abcam, ab7800, LL002, 1:100
IF, Anti-Von Willebrand Factor, Abcam, ab201336, 3E2D10+VWF635, 1:500
IF, Anti-Vimentin, Abcam, ab92547, EPR3776, 1:300
FACS, anti-human CD3, Biolegend, 300316, HIT3a, 3:100
FACS, anti-human CD19, Biolegend, 302215, HIB19, 3:100
FACS, anti-human CD19, Biolegend, 302218, HIB19, 3:100
FACS, anti-human CD68, Biolegend, 333806, Y1/82A, 3:100

Validation

All of the antibodies used in this study were validated for use in human specimens by the manufacturers and also by other researchers, with validation procedures described on the following websites of the manufactures:
Anti-alpha smooth muscle Actin: https://www.abcam.cn/alpha-smooth-muscle-actin-antibody-e184-ab32575.html

Anti-TAGLN/Transgelin/SM22: https://www.abcam.cn/taglntransgelin-antibody-ab10135.html

Anti-Cytokeratin 14: https://www.abcam.cn/cytokeratin-14-antibody-ll002-ab7800.html

Anti-Von Willebrand Factor: https://www.abcam.cn/von-willebrand-factor-antibody-3e2d10--vwf635-ab201336.html

Anti-Vimentin: https://www.abcam.cn/vimentin-antibody-epr3776-cytoskeleton-marker-ab92547.html

anti-human CD3: https://www.biolegend.com/en-us/products/pe-cyanine7-anti-human-cd3-antibody-1913

anti-human CD19: https://www.biolegend.com/en-us/products/pe-cyanine7-anti-human-cd19-antibody-1911

anti-human CD19: https://www.biolegend.com/en-us/products/apc-cyanine7-anti-human-cd19-antibody-1910

anti-human CD68: https://www.biolegend.com/en-us/products/fitc-anti-human-cd68-antibody-4844

# Human research participants

Policy information about studies involving human research participants

Population characteristics

Postmenopausal women undergoing hysterectomy surgery for POP and other benign indications were enrolled. Women undergoing hysterectomy for benign gynaecological reasons and without prolapse were enrolled as control groups. Detailed information can be found in the Patient samples section of Methods and Supplementary data 1.

Recruitment

The patients with POP and control patients were consecutively recruited between May 2018 and December 2019 at Peking Union Medical College Hospital.

Ethics oversight

The study was approved by the Ethics Committee of Peking Union Medical College Hospital.

Note that full information on the approval of the study protocol must also be provided in the manuscript.

# Flow Cytometry

## Plots

Confirm that:

- [x] The axis labels state the marker and fluorochrome used (e.g. CD4-FITC).
- [x] The axis scales are clearly visible. Include numbers along axes only for bottom left plot of group (a 'group' is an analysis of identical markers).
- [x] All plots are contour plots with outliers or pseudocolor plots.
- [x] A numerical value for number of cells or percentage (with statistics) is provided.

## Methodology

Sample preparation

Fresh vaginal wall tissues were dissociated using the single cell preparation procedures. After enzymatically digestion and washing, single cells were resuspended in 100 µL Stain Buffer freshly prepared with 3 µL of each antibody. Cells were stained for 30 min on ice, then washed with PBS and resuspended at 1×10^6 cells/mL. T cells, B cells, and macrophages were investigated using the following antibody panels: CD3, CD19, CD68.

Instrument

FACS Aria ∘ flow cytometer (BD Biosciences)

Software

FlowJo software (v.10.0.7, BD Biosciences)

Cell population abundance

No sorting performed. Flow cytometry was performed for analytical purposes.

| Gating strategy | Samples were gated by morphology based on the flow cytometry FSC and SSC. The cell adhesion and debris were excluded. Antibodies of CD3, CD19, and CD68 were detected in each sample, respectively. The corresponding isotypes were used for gating as negative population. The distribution of T cells, B cells and macrophages were quantified based on CD3-, CD19-, CD68-positive cells, respectively. |
|---|---|

☒ Tick this box to confirm that a figure exemplifying the gating strategy is provided in the Supplementary Information.
